# Distinguishing clinical characteristics of central nervous system tuberculosis in immunodeficient and non-immunodeficient individuals: a 12-year retrospective study

**DOI:** 10.1186/s12941-023-00615-w

**Published:** 2023-08-07

**Authors:** Woraseth Saifon, Khemajira Karaketklang, Anupop Jitmuang

**Affiliations:** 1 Department of Medicine, Faculty of Medicine Siriraj Hospital, Mahidol University, Bangkok, Thailand; 2 Division of Infectious Diseases and Tropical Medicine, Department of Medicine, Faculty of Medicine Siriraj Hospital, Mahidol University, 2 Wanglang Road, Bangkok Noi, Bangkok, 10700 Thailand

**Keywords:** Central nervous system, Immunodeficiency, Meningitis, *Mycobacterium tuberculosis*, Tuberculosis

## Abstract

**Background:**

Central nervous system tuberculosis (CNS TB) is a severe *Mycobacterium tuberculosis* (MTB) infection. It is unclear whether a patient’s immune status alters the clinical manifestations and treatment outcomes of CNS TB.

**Methods:**

Between January 2007–December 2018, chart reviews of CNS TB, including tuberculous meningitis (TBM), tuberculoma/abscess, and TB myelitis, were made. Subjects were categorized as immunodeficient (ID) and non-immunodeficient (NID).

**Results:**

Of 310 subjects, 160 (51.6%) were in the ID group—132 (42.6%) had HIV and 28 (9.0%) had another ID, and 150 (48.4%) were in the NID group. The mean age was 43.64 ± 16.76 years, and 188 (60.6%) were male. There were 285 (91.9%) TBM, 16 (5.2%) tuberculoma/abscess, and 9 (2.9%) myelitis cases. The TBM characteristics in the ID group were younger age (*p* = 0.003), deep subcortical location of tuberculoma (p = 0.030), lower hemoglobin level (*p* < 0.001), and lower peripheral white blood cell count (*p* < 0.001). Only HIV individuals with TBM had an infection by multidrug-resistant MTB (*p* = 0.013). TBM mortality was varied by immune status —HIV 22.8%, other ID 29.6%, and NID 14.8% (*p* < 0.001). Factors significantly associated with unfavorable outcomes in TBM also differed between the HIV and NID groups.

**Conclusions:**

TBM is the most significant proportion of CNS TB. Some of the clinical characteristics of TBM, such as age, radiographic findings, hematological derangement, and mortality, including factors associated with unfavorable outcomes, differed between ID and non-ID patients.

**Supplementary Information:**

The online version contains supplementary material available at 10.1186/s12941-023-00615-w.

## Background

Central nervous system tuberculosis (CNS TB) is one of several extrapulmonary forms of life-threatening *Mycobacterium tuberculosis* (MTB) infection. Accounting for 5–10% of extrapulmonary MTB infections, it has high morbidity and mortality rates [1–3]. In addition, immunodeficient populations—especially HIV-infected individuals, organ transplant recipients, and patients receiving corticosteroids or immunosuppressive therapy—carry an increased risk of extrapulmonary MTB infection [1–3].

Diagnosing CNS TB is troublesome because it has a variety of clinical manifestations. CNS TB may be categorized into three groups: tuberculous meningitis (TBM), an intracranial space-occupying lesion (SOL) or tuberculoma/abscess, and tuberculous myelitis or spinal cord tuberculosis. CNS TB involving immunodeficient populations is challenging. One study demonstrated that even though an HIV infection did not alter the clinical manifestations of TBM, it dramatically reduced the survival rate of HIV individuals who developed TBM [2]. A recent study from China demonstrated that younger age, agricultural work, and miliary form of TB are decisive risk factors associated with TBM [4]. However, the recent study does not focus on clinical findings of CNS TB stratified by different immune statuses [4]. Meanwhile, CNS TB and its clinical findings for Thai populations have not been thoroughly evaluated. In addition, the effects of immunodeficiency on the clinical manifestations of CNS TB and its treatment outcomes in Thai populations may differ from those in other regions. Knowing the clinical manifestations in immunodeficient people may lead to early diagnoses of CNS TB and improve its treatment outcomes. We, therefore, conducted a study to compare the clinical manifestations and treatment outcomes of immunodeficient and non-immunodeficient CNS TB patients.

## Methods

### Study design

We undertook a 12-year retrospective chart review of patients hospitalized at Siriraj Hospital from 2007 to 2018. We enrolled adult subjects aged ≥ 18 diagnosed with CNS TB, comprising TBM, tuberculoma/abscess, and tuberculous myelitis, as defined by ICD-10 codes A17.0, A17.1, A17.8, and A17.9. Patients were excluded if they had an alternative diagnosis, such as bacterial meningitis, fungal meningitis, cryptococcal meningitis, viral meningoencephalitis, neurosyphilis, and eosinophilic or parasitic meningoencephalitis. In addition, the data related to demographics, clinical manifestations, disease severity based on the modified British Medical Research Council grading [5], laboratory investigations and results, any available microbiological and pathological examinations, and treatment outcomes were reviewed. Before the commencement of this research, its protocol was approved by the Scientific Ethics Committee, Siriraj Institutional Review Board (approval no. Si480/2017).

### Microbiological diagnosis of MTB

Patient samples (such as cerebrospinal fluid or CSF, respiratory samples, tissue samples, and bodily fluids) were prepared and processed by the standard protocols of the Department of Microbiology, Siriraj Hospital. Acid-fast staining used the Kinyoun method. A commercial, real-time polymerase chain reaction (PCR) was employed for MTB complex identification (Anyplex MTB/NTM, Seegene Inc., Korea). Traditional cultures utilized Löwenstein–Jensen (LJ) solid agar and an automated liquid media system (BACTEC MGIT 960; Becton–Dickinson, Franklin Lakes, NJ, USA). Anti-TB drug susceptibility was tested via the agar proportion method and determined the mycobacterial growth ratio of drug-containing and drug-free broths.

### Definitions

The subjects were categorized into immunodeficient (ID) and non-immunodeficient (NID) groups. Individuals in the ID group were members of at least one of the following classes: HIV-infected patients; patients administered prednisolone at ≥ 20 mg/day for more than four weeks (or the equivalent dose of another systemic corticosteroid) or receiving another immunosuppressive agent; patients receiving concurrent chemotherapy; and organ transplant recipients. The NID-group individuals were not documented as having any of the ID as mentioned earlier.

The cases of CNS TB were categorized into three clinical manifestations: TBM, tuberculoma/abscess, and tuberculous myelitis. The diagnostic criteria used were modified from the consensus case definition for tuberculous meningitis as follows:

### Tuberculous meningitis (TBM)

“Definite TBM” used criteria modified from the consensus case definition for tuberculous meningitis [6]. It was defined as having clinical symptoms of meningitis (such as fever, headache, or nuchal rigidity); and either cerebrospinal fluid (CSF) analysis or a CNS radiological finding suggestive of meningitis, as defined elsewhere [6]. In addition, there needed to be a positive CSF culture for MTB, a positive CSF sample for acid-fast bacilli (AFB), or a positive result for direct PCR testing for MTB. “Probable TBM” was defined as clinical manifestations and either a CSF analysis or a CNS radiological finding suggestive of meningitis. In addition, at least one must be present: suspected active pulmonary tuberculosis or miliary tuberculosis based on chest radiography; acid-fast staining, direct PCR, or any non-CSF sample culture positive; or clinical evidence of tuberculosis outside the CNS. “Possible TBM” was defined as clinical manifestations suggestive of meningitis with or without CNS radiographic change. In addition, CSF findings with at least one of the following needed to be present: CSF lymphocytosis or a predominance of lymphocytes; a CSF protein level of > 100 mg/dL; a ratio of CSF glucose to blood glucose of < 0.5; or physician-diagnosed TBM with clinical responses and recovery of neurological deficits following an anti-TB treatment, despite an absence of evidence of positive acid-fast staining, direct PCR testing, or culture.

### Tuberculoma/abscess

“Definite tuberculoma/abscess” was the presence of clinical manifestations suggestive of intracranial tuberculoma/abscess (such as fever, headache, vomiting, seizure, and/or focal neurological deficit). Also required were CNS radiological findings that revealed tuberculoma, abscess, or space-occupying lesion. Moreover, the culture from the lesion needed to be positive for MTB, acid-fast staining, or direct PCR testing, with or without caseous granulomatous inflammation being reported in any available pathological findings. “Probable tuberculoma/abscess” was defined as the presence of clinical manifestations and CNS radiological findings suggestive of tuberculoma/abscess, plus suspected active pulmonary tuberculosis or miliary tuberculosis based on chest radiography. There also needed to be acid-fast staining, direct PCR, or any non-CNS lesion culture positive; or clinical evidence of tuberculosis outside the CNS. “Possible tuberculoma/abscess” was defined as clinical manifestations and radiological findings of CNS suggestive of tuberculoma/abscess. Furthermore, physician-diagnosed tuberculoma/abscess needed to be established, with clinical responses and recovery of neurological deficits evident following an anti-TB treatment, despite an absence of evidence of positive acid-fast staining, direct PCR testing, or culture.

### Tuberculous myelitis or spinal cord TB

“Definite tuberculous myelitis” was defined as clinical radiculopathy, myelopathy, bowel and bladder involvement, and radiological findings of the spinal cord suggestive of myelitis. Also, the CSF must have MTB culture, acid-fast staining, or direct PCR testing positive, with or without caseous granulomatous inflammation being reported in any available pathological findings. “Probable tuberculous myelitis” was defined as clinical manifestations and radiological findings suggestive of myelitis with suspected active pulmonary tuberculosis or miliary tuberculosis based on chest radiography. Alternatively, there could be acid-fasting, direct PCR, or MTB culture from any samples other than CSF or spinal tissue positive; or clinical evidence of tuberculosis outside the CNS. “Possible tuberculous myelitis” was defined as clinical manifestations and radiological findings suggestive of myelitis, with or without abnormal CSF findings. Furthermore, there needed to be physician-diagnosed tuberculous myelitis, with clinical responses and recovery of neurological deficits following an anti-TB treatment, despite an absence of evidence of positive acid-fast staining, direct PCR testing, or culture.

The treatment outcomes were categorized into favorable and unfavorable outcomes. The favorable outcomes comprised “cured” (full anti-TB treatment, with a complete response of clinical and radiological manifestations at its conclusion); and “improved” (comprehensive anti-TB treatment, but with only a partial response of clinical and radiological manifestations at its decision). The three unfavorable outcomes were “treatment default” (discontinuation of the anti-TB treatment for ≥ 2 consecutive months); “failure” (persistence, relapse, or worsening of the disease after treatment; or a severe adverse reaction preventing the continuation of the treatment); and “death” (death from any cause during the treatment period).

### Sample size calculation

Thwaites et al. found that having a positive *M. tuberculosis* culture taken from a CSF sample was a discriminating factor between HIV-infected (41.7%) and non-HIV-infected patients (29.6%) [2]. Therefore, the sample size for the present study was based on comparing proportions for two independent groups at a power of 80% and a 95% confidence level. Using the nQuery program (Statistical Solutions, Boston, MA, USA), the minimum size of each group was calculated to be 245 subjects.

### Statistical analysis

Data were respectively described using the mean (± standard deviation) for normally distributed variables or median (minimum and maximum) for non-normally distributed and frequency (percentage) for continuous and categorical data. The One-way ANOVA (Bonferroni multiple comparisons) and Kruskal Wallis H test were used to compare continuous variables, whereas Chi-square and Fisher’s exact test were performed for categorical variables between HIV infection, other ID, and NID. The following variables were analyzed to determine the factors associated with overall unfavorable outcomes. Variables with a *p*-value < 0.05 on univariate analysis were further analyzed by multivariate logistic regression (Forward method) to determine the independent predictors of overall unfavorable outcomes and presented as Odds ratio (OR) (95% confidence interval [CI]). A two-tailed p-value < 0.05 was considered statistically significant for all tests performed. PASW Statistic (SPSS) 18.0 (SPSS, Inc., Chicago, IL, USA) was used to perform all statistical analyses.

## Results

### Baseline characteristics, clinical manifestations, radiological findings, CSF profiles, laboratory findings, and treatment outcomes of all cases with CNS TB

**Fig. 1:**
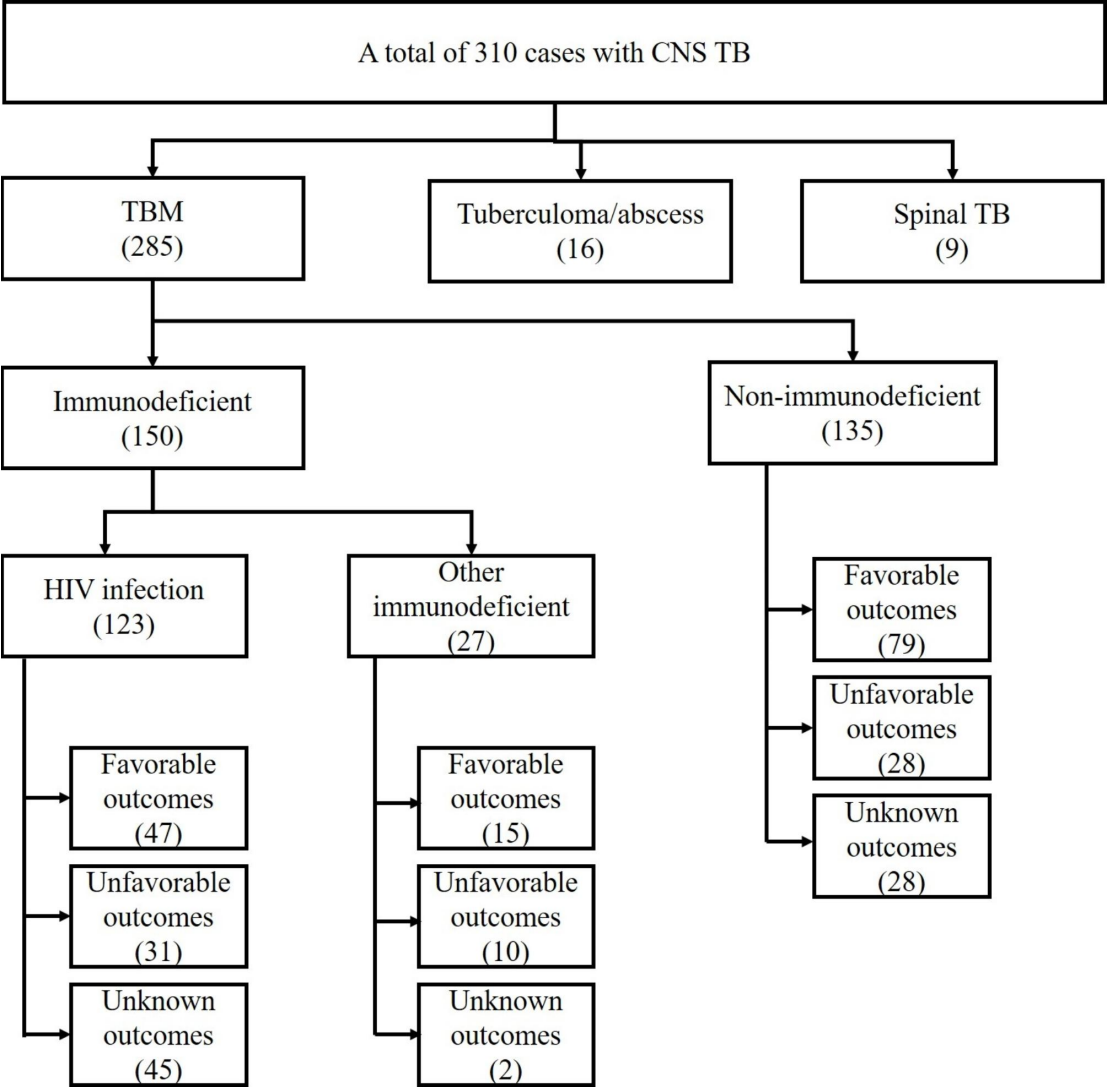
Study workflow

A total of 310 patients met the CNS TB diagnosis criteria, with 285 (91.9%) having TBM, 16 (5.2%) having tuberculoma/abscess, and 9 (2.9%) having tuberculous myelitis, as shown in Table 1; Fig. 1. The ID group had 160 (51.6%) members, while the NID group had 150 (48.4%). The leading ID conditions were HIV infections (42.6%) with a median CD4 cell level of 81 cells/mm^3^ and systemic lupus erythematosus (SLE; 7.4%). Overall, the diagnoses were stratified into 132 (42.6%) definite TB, 46 (14.8%) probable TB, and 132 (42.6%) possible TB patients. Table 1 demonstrates the imperative clinical, radiological, and laboratory findings and treatment outcomes of all 310 cases with CNS TB. CNS TB affected males more than females, mainly middle-aged adults (mean age, 43.64 years). Small proportions of CNS TB patients (24.5%) had previously documented TB. Regarding the CNS TB classified by the site of CNS involvement, 285 (91.9%) were TBM (42.5% definite, 14.4% probable, and 43.2% possible diagnoses); 16 (5.2%) were tuberculoma/abscess (50.0% definite, 31.2% probable, and 18.8% possible diagnoses); and 9 (2.9%) were myelitis (33.3% definite and 66.7% possible diagnoses). Pulmonary TB (33.2%) and TB lymphadenitis (6.1%) were the most common sites concurrently found at the onset. The median duration of symptoms was 14 days. Fever, headache, signs of meningeal irritation, and impaired cognitive function were common clinical manifestations of CNS TB. According to the modified British Medical Research Council grading, 30 (9.7%) patients initially presented with a severity grade of 3 at the onset. Computed tomography (CT) and magnetic resonance imaging (MRI) exhibited several radiological findings: abnormal meningeal enhancement (49.7%); hydrocephalus (32.7%); and cerebral infarction (28.9%); and single or multiple tuberculomas or abscesses, at various sites (14.1%). The overall profile of the CSF findings revealed increased numbers of white blood cells (median, 120 cells/mm3), with lymphocytes predominating (median, 80%), elevated protein levels (median, 177.5 mg/dL), and reduced glucose levels (median, 36.0 mg/dL), including the low CSF/blood glucose ratio (median, 0.31). The microbiological diagnoses of CNS TB in overall patients were mainly derived from positive MTB cultures from CSF or tissue sections; acid-fast staining and direct PCR testing gave positive results in smaller numbers. Of the 310 patients, MTB isolated from CSF, CNS tissue sections, and extra-CNS sites of 128 patients (41.3%) were tested for anti-TB drug susceptibility. Most patients (79.7%) had MTB isolates fully susceptible to the tested anti-TB drugs; 8.6% had MTB resistance to isoniazid alone, while 3.9% had MTB that exhibited multidrug resistance. Most patients (74.8%) received a standard combination regimen of isoniazid, rifampicin, pyrazinamide, and ethambutol for the initial anti-TB treatment. Only 25.2% of patients required alternative regimens due to several factors (Table 1). Two hundred (64.5%) patients received adjunctive corticosteroid therapy. Of 110 patients who did not receive the adjunctive steroid therapy, only 30 cases had medical notes well documented the reasons for the underuse of this agent, such as physician preferences 18 cases, concern for complications of more immunosuppression effect 7 cases, and having other opportunistic infections 5 cases. Unfortunately, the documented reasons for underusing steroid therapy were unavailable in most patients (80). A range of surgical interventions was performed on 51 patients (16.5%) because of the development of complications from CNS TB. Unfortunately, 85 (27.4%) of the patients had unknown outcomes at the end of treatment due to loss of follow-up, transfer to other facilities or unavailable data. Of the remaining 225 patients with known outcomes, 155 (68.9%) had favorable outcomes, while 70 (31.1%) had unfavorable outcomes, and 56 (18.1) had fatal outcomes.

**Table 1:** Baseline characteristics, clinical manifestations, radiological findings, CSF profiles, laboratory findings, and treatment outcomes of all 310 cases with CNS TB

Variable	Total (n = 310)	Variable	Total (n = 310)
Male, n (%)	188 (60.6)	**CNS CT or MRI findings**, n (%) cont.	
Age, mean ± SD, years	43.64 ± 16.76	Location of tuberculoma/abscess	
**The final diagnosis of CNS TB**, n (%)		Grey-white matter junction	31 (75.6)
Definite	132 (42.6)	Deep subcortical location	17 (41.5)
Probable	46 (14.8)	Brainstem	10 (24.4)
Possible	132 (42.6)	Cerebellum	9 (22.0)
BMI, mean ± SD, kg/m²	20.42 ± 3.62	Spinal cord	9 (2.9)
Previous TB diagnosis, n (%)	76 (24.5)	Cerebral infarction	86 (28.9)
**ID conditions**, n (%)		**CSF findings**	
HIV infection	132 (42.6)	OP, mean ± SD, cmH_2_O	21.83 ± 9.50
CD_4_ cell level, median (min-max), cells/mm^3^	81.0 (37.0–170.0)	WBC count, median (min-max), cells/mm^3^	120.0 (0.0–2,800)
SLE	23 (7.4)	%neutrophils, median (min-max), %	14.0 (0.0–99)
Others	5 (1.6)	%lymphocytes, median (min-max), %	80.0 (0.0–100)
NID, n (%)	150 (48.4)	Protein, median (min-max), mg/dL	177.5 (12–5,877)
**Other comorbidities**, n (%)		Glucose, median (min-max), mg/dL	36.0 (1.0–141)
DM	19 (6.1)	CSF to plasma glucose ratio, median (min-max)	0.31 (0.01–0.84)
HT	41 (13.2)	AFB positive, n (%)	8 (2.7)
Kidney disease	10 (3.2)	Direct PCR MTB positive, n (%)	47 (15.7)
Liver disease	17 (5.5)	MTB culture positive, n (%)	112 (37.5)
Heart disease	9 (2.6)	**CNS tissue pathology examination**, n (%)	
Lung disease	37 (11.9)	AFB positive	11 (39.3)
Cancers in remission	9 (2.9)	Direct PCR MTB positive	9 (39.1)
Others	35 (11.3)	MTB culture positive	14 (51.9)
**Type and final diagnosis of CNS TB**		**Initial laboratory testing**	
**TBM**, n (%)		Hb, mean ± SD, g/dL	11.47 ± 2.22
Definite	121 (42.5)	Hct, mean ± SD, percent	34.86 ± 6.44
Probable	41 (14.4)	WBC count, median (min-max), cells/mm^3^	7,865 (900–39,950)
Possible	123 (43.2)	%neutrophils, mean ± SD, %	73.69 ± 14.63
**Tuberculoma/abscess**^a^, n (%)		%lymphocytes, median (min-max), %	13.0 (0.0–83.0)
Definite	8 (50.0)	BUN, median (min-max), mg/dL	13.0 (0.8–136)
Probable	5 (31.2)	Cr, median (min-max), mg/dL	0.78 (0.30–9.30)
Possible	3 (18.8)	AST, median (min-max), U/L	29.0 (9.0–1,056)
**Myelitis**, n (%)		ALT, median (min-max), U/L	23.0 (2.0–788)
Definite	3 (33.3)	ALP, median (min-max), IU/L	79.0 (24.0–706)
Probable	0 (0.0)	Albumin, mean ± SD, g/dL	3.42 ± 0.69
Possible	6 (66.7)	Sodium level, mean ± SD, mmol/L	131.65 ± 6.68
**Concurrent active non-CNS TB**, n (%)		**Anti-TB drug susceptibility testing**^c^, n (%)	
Lung	103 (33.2)	Performed	128 (41.3)
Pleura	2 (0.6)	Not performed	182 (58.7)
Lymph node	19 (6.1)	Fully susceptible	102 (79.7)
Others	19 (6.1)	Isoniazid monoresistance	11 (8.6)
Median onset of symptoms, days (min-max)	14.0 (1.0–210)	Rifampin monoresistance	2 (1.6)
**Clinical manifestations**, n (%)		Pyrazinamide monoresistance	5 (3.9)
Fever	194 (62.6)	Multidrug resistance	5 (3.9)
Headache	170 (54.8)	**Initial anti-TB treatment**, n (%)	
Vomiting	99 (31.9)	Standard combination regimen^d^	232 (74.8)
Meningeal irritation signs	205 (66.1)	Alternative or modified regimen^e^	78 (25.2)
Impaired cognitive function	143 (46.1)	Adjunctive corticosteroid therapy, n (%)	200 (64.5)
Seizure	39 (12.6)	**Surgical interventions**, n (%)	
Hemiparesis	30 (9.7)	Temporary ventriculostomy	21 (6.8)
Paraparesis	9 (2.9)	Ventriculoperitoneal shunt	2 (0.6)
Multi-cranial nerve palsy	13 (26.5)	Vertebral laminectomy	10 (3.2)
Abnormal movement	6 (1.9)	Vertebral corpectomy	1 (0.3)
Impaired sensory systems	20 (6.5)	Burr hole	2 (0.6)
Bowel and bladder dysfunctions	21 (6.8)	Others	15 (4.8)
Cerebellar signs	18 (5.8)	**Outcomes at the end of treatment**, n (%)	
Abnormal gait	10 (3.2)	Death	56 (18.1)
GCS score, mean ± SD	13.62 ± 2.30	Cure	51 (16.5)
Modified BMRC TBM grade III^b^, n (%)	30 (9.7)	Improvement	104 (33.5)
**CNS CT or MRI findings**, n (%)		Failure	9 (2.9)
Meningeal enhancement	149 (49.7)	Default^f^	5 (1.6)
Hydrocephalus	98 (32.7)	Unknown^g^	85 (27.4)
Tuberculoma/abscess	43 (14.1)	**Treatment outcomes**, n (%)	
Single lesion	14 (32.6)	Favorable	155 (68.9)
Multiple lesions	29 (67.4)	Unfavorable	70 (31.1)

Abbreviations: %lymphocyte, percentage of lymphocytes; %neutrophil, percentage of neutrophils; AFB, acid-fast bacilli; ALP, alkaline phosphatase; ALT, alanine transaminase; AST, aspartate transaminase; BMI, body mass index; BMRC, British Medical Research Council; BUN, blood urea nitrogen; CN, cranial nerve; CNS, central nervous system; Cr, creatinine; CSF, cerebrospinal fluid; CT, computed tomography; DM, diabetes mellitus; GCS, Glasgow Coma Scale; Hb, hemoglobin; Hct, hematocrit; HIV, human immunodeficiency virus; HT, hypertension; MRI, magnetic resonance imaging; MTB, *Mycobacterium tuberculosis*; NID, non-immunodeficiency; OP, opening pressure; PCR, polymerase chain reaction; SLE, systemic lupus erythematosus; TB, tuberculosis; TBM, tuberculous meningitis; WBC, white blood cell

^a^ Clinical findings suggestive of a predominately intracranial space-occupying lesion, such as tuberculoma or abscess with no evidence of TB meningitis

^b^ Defined as disease severity with a GCS score ≤ 10

^c^ Anti-TB drug susceptibility was tested by the agar proportion method and the determination of the mycobacterial growth ratio of drug-containing and drug-free broths

^d^ A combination of anti-TB agents, namely, isoniazid, rifampin, pyrazinamide, and ethambutol

^e^ A standard anti-TB regimen was switched or modified to alternative agents due to adverse reactions, drug intolerance, or drug allergy

^f^ Due to several reasons, such as poor adherence, lack of caregiver, having a new job or housing in other provinces, and patient unawareness

^g^ Due to several reasons, such as loss of follow-up, transfer to other facilities, and unavailable data

### Comparison of clinical characteristics, radiological and laboratory findings, and treatment outcomes of tuberculous meningitis between patients with and without immunodeficiency

TBM (285) was the large proportion of CNS TB diagnoses, while a small proportion of diagnoses were tuberculoma/abscess (16) and tuberculous myelitis (9). Therefore, we included 285 patients with TBM to analyze clinical characteristics, radiological and laboratory findings, and treatment outcomes. Of 285 TBM patients, they were classified into the HIV group (123), another ID group (27), and the NID group (135), as shown in Fig. 1; Table 2. Our study demonstrated there were several baseline characteristics significantly different between those 3 groups, such as gender (p < 0.001), age (p = 0.003), hypertension (p < 0.001), heart disease (p = 0.014), lung disease (p = 0.007), and kidney disease (p = 0.048). In addition, tuberculous lymphadenitis was concurrent tuberculosis more commonly found in the HIV group than in other groups (p = 0.004). The onset of symptoms, clinical manifestations, and radiological findings of TBM were comparable between the three groups; however, the ID groups (HIV and another ID) had a more significant proportion of coincidental findings of tuberculoma or abscess in the deep subcortical area detected by the cranial imaging (p = 0.030). Most of the CSF findings, such as the opening pressure, number of white blood cells, protein, and glucose levels, were comparable between the three groups. However, patients in another ID group had a significantly higher percentage of CSF neutrophils (p = 0.042) but a significantly lower percentage of CSF lymphocytes (p = 0.018) than patients in other groups. The microbiological diagnoses of TBM in all groups were mainly derived from positive CSF cultures (p = 0.189). Still, CSF samples from other ID groups gave a greater positive result of the direct PCR test (p = 0.006) than in other groups. For the initial laboratory testing, there were several hematological and chemistry testing that showed a statistically significant difference in results among 3 groups, such as hemoglobin level (p < 0.001), percentage of hematocrit (p < 0.001), number of peripheral white blood cell counts (p < 0.001), percentage of peripheral neutrophils (p = 0.020), aspartate transaminase (p = 0.003) and alanine transaminase (p = 0.043) levels, and albumin level (p = 0.002). Anti-TB drug susceptibility was performed on 119 MTB isolated from the patient’s samples (41.8%). Patients in the NID group (85.9%) seemed to have more prevalence of the MTB isolates fully susceptible to the anti-TB drugs than those in the HIV (69.6%) and another ID (66.7%) groups (p = 0.084). In addition, the HIV group had multidrug-resistant (MDR) isolates (10.9%), while another ID and the NID groups had no MDR isolates detected (p = 0.013). Most of the ID and NID patients received a standard combination regimen for TBM management. Patients in the HIV group received significantly fewer adjunctive corticosteroid therapy than patients in another ID and the NID groups (p < 0.001). A few patients underwent temporary ventriculostomy, which was more common in other ID and the NID groups than in the HIV group (p = 0.011). Our study demonstrated that 36.6% of the HIV, 20.7% of the NID, and 7.4% of other ID groups had unknown outcomes (Table 2). Patients in the HIV and other ID groups had more fatal outcomes, whereas the HIV group had fewer cures and improvement outcomes (p < 0.001). Based on those findings, we considered immunodeficiency’s effects may alter TBM’s clinical features and outcomes. Thus, we analyzed the factors determining unfavorable outcomes separately between HIV and NID populations.

**Table 2:** Comparison of baseline characteristics, clinical manifestations, radiological findings, CSF profiles, laboratory findings, and treatment outcomes of tuberculous meningitis between patients with and without immunodeficiency (n = 285)^a^

Variable	HIV infection (n = 123)	Other ID (n = 27)	NID (n = 135)	*p-*value
Male, n (%)	87 (70.7)	4 (14.8)	79 (58.5)	< 0.001^*^
Age, mean ± SD, years	40.65 ± 10.99	40.15 ± 14.42	47.81 ± 20.84	0.003^*^
BMI, mean ± SD, kg/m²	19.74 ± 3.68	20.25 ± 2.59	20.61 ± 3.56	0.282
Previous TB diagnosis, n (%)	42 (34.1)	0 (0.0)	26 (19.3)	< 0.001^*^
**Other comorbidities**, n (%)	123	27	135	
DM	3 (2.4)	2 (7.4)	13 (9.6)	0.058
HT	4 (3.3)	5 (18.5)	30 (22.2)	< 0.001^*^
Kidney disease	1 (0.8)	2 (7.4)	7 (5.2)	0.048^*^
Liver disease	9 (7.3)	1 (3.7)	5 (3.7)	0.400
Heart disease	0 (0.0)	1 (3.7)	8 (5.9)	0.014^*^
Lung disease	22 (17.9)	0 (0.0)	11 (8.1)	0.007^*^
Cancers in remission	2 (1.6)	1 (3.7)	6 (4.4)	0.389
Others	11 (8.9)	3 (11.1)	20 (14.8)	0.345
**The final diagnosis of TBM**, n (%)				0.237
Definite	44 (35.8)	12 (44.4)	65 (48.1)	
Probable	22 (17.9)	2 (7.4)	17 (12.6)	
Possible	57 (46.3)	13 (48.1)	53 (39.3)	
**Concurrent active non-CNS TB**, n (%)				
Lung	47 (38.2)	7 (25.9)	44 (32.6)	0.397
Pleura	1 (0.8)	0 (0.0)	1 (0.7)	1.00
Lymph node	15 (12.2)	0 (0.0)	4 (3.0)	0.004^*^
Others	5 (4.1)	3 (11.1)	11 (8.1)	0.263
Median onset of symptoms, median (min-max), days	10.0 (1.0-168.0)	14.0 (1.0–84.0)	14.0 (1.0-140.0)	0.297
**Clinical manifestations**, n (%)				
Fever	77 (62.6)	16 (59.3)	83 (61.5)	0.945
Headache	73 (59.3)	16 (59.3)	70 (51.9)	0.447
Vomiting	38 (30.9)	8 (29.6)	48 (35.6)	0.676
Meningeal irritation signs	85 (69.1)	20 (74.1)	86 (63.7)	0.467
Impaired cognitive function	60 (48.8)	15 (55.6)	60 (44.4)	0.525
Seizure	12 (9.8)	4 (14.8)	16 (11.9)	0.716
Hemiparesis	16 (13.0)	3 (11.1)	8 (5.9)	0.145
Paraparesis	0 (0.0)	0 (0.0)	0 (0.0)	-
Multi-cranial nerve palsy	3/18 (16.7)	2/4 (50.0)	6/21 (28.6)	0.293
Abnormal movement	3 (2.4)	1 (3.7)	2 (1.5)	0.456
Impaired sensory systems	7 (5.7)	1 (3.7)	4 (3.0)	0.547
Bowel and bladder dysfunctions	6 (4.9)	0 (0.0)	4 (3.0)	0.558
Cerebellar signs	6 (4.9)	0 (0.0)	9 (6.7)	0.355
Abnormal gait	4 (3.3)	1 (3.7)	3 (2.2)	0.659
GCS score, mean ± SD	13.33 ± 2.48	13.70 ± 1.92	13.70 ± 2.32	0.420
Modified BMRC TBM grade III^b^, n (%)	18 (14.6)	2 (7.4)	10 (7.4)	0.144
**CNS CT or MRI findings**, n (%)				
Meningeal enhancement	60 (49.2)	10 (38.5)	68 (51.9)	0.455
Hydrocephalus	40 (32.8)	9 (34.6)	43 (32.8)	0.983
Tuberculoma/abscess	11	1	15	
Single lesion	1 (9.1)	0 (0.0)	6 (40.0)	0.222
Multiple lesions	10 (90.9)	1 (100.0)	9 (60.0)	
Location of tuberculoma/abscess	11	1	15	
Grey-white matter junction	8/11 (72.7)	1/1 (100.0)	14/15 (93.3)	0.386
Deep subcortical location	8/11 (72.7)	1/1 (100.0)	4/15 (26.7)	0.030^*^
Brainstem	3/11 (27.3)	1/1 (100.0)	4/15 (26.7)	0.450
Cerebellum	2/11 (18.2)	0/1 (0.0)	5/15 (33.3)	0.746
Spinal cord	1/123 (0.8)	0/27 (0.0)	2/135 (1.5)	1.00
Cerebral infarction	47/122 (38.5)	7/26 (26.9)	32/130 (24.6)	0.052
**CSF findings**	122	27	132	
OP, mean ± SD, cmH_2_O	21.44 ± 10.13	23.81 ± 9.50	21.77 ± 8.91	0.538
WBC count, median (min-max), cells/mm^3^	99.50 (0.0-1500)	78.0 (8.0-1311)	140 (2.0-2800)	0.343
%neutrophils, mean ± SD, %	27.09 ± 29.78	43.36 ± 34.94	24.26 ± 26.72	0.042^*^
%lymphocytes, median (min-max), %	79.0 (0-100)	46.0 (7.0-100)	83.0 (1.0-100)	0.018^*^
Protein, median (min-max), mg/dL	187 (20-5877)	168 (42–499)	174.5 (40.3–4482)	0.836
Glucose, median (min-max), mg/dL	37 (2-122)	27 (4.3–88)	36 (1-141)	0.304
CSF to plasma glucose ratio, median (min- max)	0.30 (0.02–0.84)	0.25 (0.04–0.50)	0.32 (0.1–0.78)	0.343
AFB positive, n (%)	4/122 (3.3)	0/27 (0.0)	4/132 (3.0)	1.00
Direct PCR MTB positive, n (%)	19/122 (15.6)	10/27 (37.0)	16/132 (12.1)	0.006^*^
MTB culture positive, n (%)	44/122 (36.1)	7/27 (25.9)	57/132 (43.2)	0.189
**CNS tissue pathology examination**, n (%)	4	0	10	
AFB positive	1/4 (25.0)	0 (0.0)	3/10 (30.0)	1.00
Direct PCR MTB positive	0/3 (0.0)	0 (0.0)	4/9 (44.4)	0.491
MTB culture positive	3/3 (100.0)	0 (0.0)	3/9 (33.3)	0.182
**Initial laboratory testing**				
Hb, mean ± SD, g/dL	10.83 ± 2.32	10.77 ± 1.92	12.02 ± 2.02	< 0.001^*^
Hct, mean ± SD, percent	32.86 ± 6.73	33.17 ± 6.24	36.43 ± 5.68	< 0.001^*^
WBC count, median (min-max), cells/mm^3^	6900 (1140–28,290)	6520 (900-13990)	8980 (3860–39,950)	< 0.001
%neutrophils, mean ± SD, %	70.93 ± 16.88	74.06 ± 16.24	76.28 ± 12.15	0.020^*^
%lymphocytes, median (min-max), %	14.0 (1.0–60.0)	14.0 (0.0–60.0)	12.0 (2.3–83.0)	0.129
BUN, median (min-max), mg/dL	13.8 (0.8–136.0)	13.8 (2.7–80.7)	12.9 (2.2–95.0)	0.994
Cr, median (min-max), mg/dL	0.8 (0.3–5.1)	0.8 (0.4–2.5)	0.7 (0.3–9.3)	0.484
AST, median (min-max), U/L	36.0 (12.0-1056)	27.0 (11.0-226)	24.0 (9.0-279.0)	0.003^*^
ALT, median (min-max), U/L	25.0 (4.0-516.0)	18.0 (4.0–75.0)	23.0 (2.0-788.0)	0.043^*^
ALP, median (min-max), IU/L	86.0 (25.0-706.0)	84.0 (45.0-340.0)	75.0 (25.0-428.0)	0.097
Albumin, mean ± SD, g/dL	3.31 ± 0.69	3.09 ± 0.59	3.53 ± 0.66	0.002^*^
Sodium level, mean ± SD, mmol/L	130.77 ± 6.55	133.48 ± 5.15	131.62 ± 7.01	0.147
**Anti-TB drug susceptibility testing**^c^, n (%)				0.172
Performed	46 (37.4)	9 (33.3)	64 (47.4)	
Not performed	77 (62.6)	18 (66.7)	71 (52.6)	
Fully susceptible	32/46 (69.6)	6/9 (66.7)	55/64 (85.9)	0.084
Isoniazid monoresistance	6/46 (13.0)	2/9 (22.2)	3/64 (4.7)	0.099
Rifampin monoresistance	1/46 (2.2)	0/9 (0.0)	1/64 (1.6)	1.00
Pyrazinamide monoresistance	1/46 (2.2)	0/9 (0.0)	4/64 (6.2)	0.596
Multidrug resistance	5/46 (10.9)	0/9 (0.0)	0/64 (0.0)	0.013
**Initial anti-TB treatment**, n (%)				0.520
Standard combination regimen^d^	93 (75.6)	18 (66.7)	104 (77.0)	
Alternative or modified regimen^e^	30 (24.4)	9 (33.3)	31 (23.0)	
Adjunctive corticosteroid therapy, n (%)	65 (52.8)	22 (81.5)	101 (74.8)	< 0.001^*^
**Surgical interventions**, n (%)	123	27	135	
Temporary ventriculostomy	2 (1.6)	4 (14.8)	11 (8.1)	0.011^*^
Ventriculoperitoneal shunt	0 (0.0)	1 (3.7)	1 (0.7)	0.181
**Outcomes at the end of treatment**, n (%)	123	27	135	
Death	28 (22.8)	8 (29.6)	20 (14.8)	< 0.001^*^
Cure	13 (10.6)	7 (25.9)	31 (23.0)	
Improvement	34 (27.6)	8 (29.6)	48 (35.6)	
Failure	3 (2.4)	0 (0.0)	6 (4.4)	
Default	0 (0.0)	2 (7.4)	2 (1.5)	
Unknown	45 (36.6)	2 (7.4)	28 (20.7)	
**Treatment outcomes**, n (%)	78	25	107	
Favorable	47 (60.3)	15 (60.0)	79 (73.8)	0.109
Unfavorable	31 (39.7)	10 (40.0)	28 (26.2)	

Abbreviations: %lymphocyte, percentage of lymphocytes; %neutrophil, percentage of neutrophils; AFB, acid-fast bacilli; ALP, alkaline phosphatase; ALT, alanine transaminase; AST, aspartate transaminase; BMI, body mass index; BMRC, British Medical Research Council; BUN, blood urea nitrogen; CN, cranial nerve; CNS, central nervous system; Cr, creatinine; CSF, cerebrospinal fluid; CT, computed tomography; DM, diabetes mellitus; GCS, Glasgow Coma Scale; Hb, hemoglobin; Hct, hematocrit; HIV, human immunodeficiency virus; HT, hypertension; MRI, magnetic resonance imaging; MTB, *Mycobacterium tuberculosis*; NID, non-immunodeficiency; OP, opening pressure; PCR, polymerase chain reaction; TB, tuberculosis; TBM, tuberculous meningitis; SLE, systemic lupus erythematosus; WBC, white blood cell

^a^ Excluding patients with tuberculoma/abscess (16) and tuberculous myelitis (9)

^b^ Defined as disease severity with a GCS score ≤ 10

^c^ Anti-TB drug susceptibility was tested by the agar proportion method and the determination of the mycobacterial growth ratio of drug-containing and drug-free broths

^d^ A combination of anti-TB agents, namely, isoniazid, rifampin, pyrazinamide, and ethambutol

^e^ A standard anti-TB regimen was switched or modified to alternative agents due to adverse reactions, drug intolerance, or drug allergy

* *p* < 0.05; multiple comparisons of mean with Bonferroni or Game-Howell comparison

### Factors associated with overall unfavorable outcomes in HIV-infected patients with tuberculous meningitis

After excluding 45 TBM patients with unknown outcomes, 78 patients were available to evaluate the treatment outcomes: 47 had favorable outcomes, and 31 had unfavorable outcomes (Supplementary Table 1). After adjusting for several variables, the multivariate analysis identified several factors as still significantly associated with unfavorable outcomes for TBM in HIV-infected patients (Table 3). They were modified BMRC TBM grade III (AOR, 20.51; 95% CI, 2.10–200.40; p = 0.009); a radiological finding of meningeal enhancement (AOR, 5.75; 95% CI, 1.63–20.27; p = 0.007); and a low percentage of peripheral blood lymphocytes (AOR, 0.87; 95% CI, 0.81–0.94; p = 0.001).

**Table 3:** Factors associated with overall unfavorable outcomes in HIV-infected patients with tuberculous meningitis

Factor	Univariate		Multivariate	
	OR (95% CI)	*p-*value	OR (95% CI)	*p-*value
The final diagnosis of TBM				
Definite	1		-	-
Probable	1.73 (0.44–6.79)	0.430	-	-
Possible	0.41 (0.14–1.17)	0.096	-	-
**Clinical characteristics**				
BMI	0.72 (0.53–0.96)	0.026	-	-
Liver disease	3.33 (0.57–19.44)	0.181	-	-
Impaired cognitive function	2.33 (0.92–5.90)	0.074	-	-
GCS score	0.64 (0.50–0.83)	0.001	-	-
Modified BMRC TBM grade III	7.83 (1.54–39.90)	0.013	20.51 (2.10-200.40)	0.009
CD_4_ cell count level	0.99 (0.98–0.99)	0.022	-	-
**CNS CT or MRI findings**				
Meningeal enhancement	2.84 (1.10–7.33)	0.031	5.75 (1.63–20.27)	0.007
Hydrocephalus	3.54 (1.35–9.29)	0.010	-	-
Cerebral infarction	2.73 (1.05–7.16)	0.040	-	-
**CSF findings**				
%neutrophils	1.02 (1.001–1.04)	0.039	-	-
%lymphocytes	0.99 (0.97-1.00)	0.054	-	-
Direct PCR MTB positive, n (%)	3.91 (1.06–14.42)	0.041	-	-
**Initial laboratory testing**				
WBC count	1.001 (0.99–1.002)	0.108	-	-
%neutrophils	1.04 (1.01–1.08)	0.017	-	-
%lymphocytes	0.90 (0.85–0.96)	0.001	0.87 (0.81–0.94)	0.001
Cr	0.56 (0.13–2.44)	0.440	-	-
Albumin	0.59 (0.32–1.08)	0.088	-	-

Abbreviations: BMI, body mass index; BMRC, British Medical Research Council; CNS, central nervous system; Cr, creatinine; CSF, cerebrospinal fluid; CT, computerized tomography; GCS, Glasgow Coma Scale; MRI, magnetic resonance imaging; MTB, *Mycobacterium tuberculosis*; PCR, polymerase chain reaction; OR, odds ratio; TBM, tuberculous meningitis; WBC, white blood cell

### Factors associated with overall unfavorable outcomes in non-immunodeficient patients with tuberculous meningitis

After excluding 28 TBM patients with unknown outcomes, 107 patients were available to evaluate the treatment outcomes: 79 had favorable outcomes, and 28 had unfavorable outcomes (Supplementary Table 2). After adjusting for several variables, the multivariate analysis identified several factors as still significantly associated with unfavorable outcomes for TBM in NID patients (Table 4). They were a reduced Glasgow Coma Scale score (AOR, 0.62; 95% CI, 0.49–0.80; p < 0.001); a radiological finding of cerebral infarction (AOR, 4.18; 95% CI, 1.03–14.49; p = 0.024); the underuse of adjunctive corticosteroid therapy (AOR, 0.28; 95% CI, 0.08–0.93; p = 0.037); and an undergoing temporary ventriculostomy (AOR, 9.67; 95% CI, 1.09–85.91; p = 0.042).

**Table 4:** Factors associated with overall unfavorable outcomes in non-immunodeficient patients with tuberculous meningitis

Factor	Univariate		Multivariate	
	Crude OR (95% CI)	*p-*value	Adjusted OR (95% CI)	*p-*value
Age	1.03(1.01–1.06)	0.003	-	-
HT	4.00(1.49–10.77)	0.006	-	-
Kidney disease	13.00(1.39-121.94)	0.025	-	-
Heart disease	4.22(0.88–20.21)	0.071	-	-
Other comorbidities	3.55(1.18–10.65)	0.024	-	-
Initial GCS score	0.58(0.45–0.74)	< 0.001	0.62(0.49–0.80)	< 0.001
Initial modified BMRC TBM grade III	10.50(1.98–55.72)	0.006	-	-
Cerebral infarction	2.69(1.01–7.15)	0.047	4.18(1.03–14.49)	0.024
Receiving adjunctive corticosteroid therapy	0.34(0.14–0.85)	0.020	0.28(0.08–0.93)	0.037
Temporary ventriculostomy	8.37(1.52–46.03)	0.015	9.67(1.09–85.91)	0.042

Abbreviations: BMRC, British Medical Research Council; GCS, Glasgow Coma Scale; HT, hypertension; OR, odds ratio; TBM, tuberculous meningitis

## Discussion

It has been unclear whether a patient’s immune status can alter the clinical manifestations and treatment outcomes of CNS TB. This 12-year retrospective chart review demonstrated the clinical manifestations and treatment outcomes of 160 ID (132 HIV, 28 other ID) and 150 NID patients diagnosed and hospitalized with CNS TB. The proportions of the CNS TB final diagnoses in the current study varied by the clinical manifestations. The final diagnoses for the TBM case were mainly definite and possible; only a tiny proportion of the patients were classed as having a probable diagnosis. The finding was different from a previous study, in which most of the TBM cases had definite (56%) and probable (34%) diagnoses [7]. Earlier research also demonstrated the higher rates of acid-fast smear, cultures, and molecular testing of CSF and non-CSF samples [7]. Those higher positive findings may explain why the previous research had more excellent rates for the definite and probable TBM diagnoses than ours. Regarding tuberculoma/abscess, the patients in the present work mainly had definite or probable diagnoses because they had more positive evidence of MTB infection from CNS samples or had active TB from an extra-CNS site. For tuberculous myelitis, the proportion of patients with a possible diagnosis was higher than that for a definite diagnosis. Tuberculous myelitis is rare; it is usually challenging to prove a diagnosis due to its paucibacillary MTB infection and the difficulty in obtaining tissues that provide a definite diagnosis [8, 9].

Our study demonstrated that CNS TB was frequently involved in males (60.6%) and middle age populations (mean age 43.64 years); however, the ID patients with TBM were significantly younger than the NID patients with TBM. These presentations were similar to findings from other studies [2, 10–12] since HIV infection and other ID conditions, such as SLE, are usually common in young and middle-aged adults. In addition, a previous TB diagnosis was significantly more common among HIV patients (34.1%), whereas the previously documented TB reported by other studies varied from 7.6 to 58% based on the area of low or high TB prevalence [2, 10, 13, 14]. Overall, patients with CNS TB had a lower rate of concurrent pulmonary TB (33.2%) than reported by other studies (50–70%), which included all abnormal chest radiographic findings of both active and inactive lesions or old lesions [15, 16]. Our work only included patients with clinically or radiologically documented active pulmonary disease, which may have limited the rate of concurrent pulmonary TB. Contemporary TB of the lymph nodes was the extra-CNS manifestation more commonly found in the HIV group, consistent with other research [2].

The clinical manifestations of TBM were not significantly different between the ID and NID groups. In addition, the median duration of symptoms in each group was subacute onset, similar to previous studies [2, 17]. Meningeal enhancement, hydrocephalus, and cerebral infarction were the most frequent radiographic abnormalities in CT and MRI studies. These findings are similar to a large neuroimaging study [18]. In contrast, CNS tuberculoma by imaging studies was found less frequently in the present work than in the previous study [18]. At our institute, CT scanning generally prioritizes MRI analysis for the initial diagnoses of patients with suspected CNS TB. Therefore, many study patients did not receive serial or subsequent MRI, which might cause missing an early or small CNS lesion. Our study found that a radiological finding of tuberculoma/abscess, particularly in the deep subcortical areas, occurred more frequently in the HIV group than in the NID group. This finding is atypical for CNS tuberculomas and abscesses, usually located at the grey-white matter junction and the periventricular areas [19, 20].

Similar to previous studies, the CSF profiles of the overall patients in the current investigation showed typical but non-specific features, such as mildly elevated opening pressures, CSF pleocytosis with lymphocytes predominating, elevated protein levels, and low glucose levels [2, 7, 21, 22]. However, CSF profiles of TBM in another ID group had a significantly lower percentage of CSF lymphocytes and a more significant percentage of CSF neutrophils than other groups (Table 2). The CSF profile without lymphocyte predominated was an unusual CSF finding for TBM, which still has been puzzling in our study. Several studies reported that subacute meningitis without lymphocyte-predominant CSF might be attributable to an early onset of TBM, nocardiosis, fungal infection, enterovirus infection, and herpes virus infection [23, 24].

Standard diagnostic testing—such as acid-fast staining, direct PCR, and CSF culturing, gave a lower positive yield. Our results have concordance with previous studies that showed low diagnostic sensitivities of acid-fast staining (11–34%), Xpert MTB/rifampicin (25%), and mycobacterial cultures (32–44%) when using clinical diagnostic gold standards [25, 26]. Our findings demonstrated that CSF and tissue sampling for mycobacterial cultures were the most crucial diagnostic testing for CNS TB since they exhibited greater diagnostic yields than direct PCR testing and acid-fast staining method (Table 1). A high CSF volume was an independent factor associated with microbiologically confirmed TBM [26]. Surprisingly, another ID group had a more significant proportion of direct PCR testing positive (37.0%), but the CSF cultures gave 25.9% MTB positive results in this group (Table 2). Inadequate CSF sampling, a low mycobacterial load, or false positive PCR testing may cause this discordant result. However, we recommend that an anti-TB administration should not be delayed after excluding other possible causes when the direct PCR MTB testing is positive in an ID individual with suspected TBM.

As to the MTB isolates, the overall rates of isoniazid monoresistance and multidrug resistance in CNS TB were relatively low, as was reported by another study [27]. The rates seemed comparable to the drug resistance rate observed for pulmonary TB in Thai patients [28, 29]. However, the patients in the HIV group appeared to have a lower rate of first-line anti-TB drug susceptibility. In contrast, they had a significantly higher rate of multidrug-resistant TB. This group corresponds with the findings of several epidemiological studies [1–3]. Reduced anti-TB susceptibility may affect the treatment outcomes of TBM in patients with an immunodeficiency disorder [30]. However, this study could not prove the association between anti-TB resistance and the outcomes since there were too small numbers of study populations. A combination of isoniazid, rifampin, pyrazinamide, and ethambutol was this study’s mainstay of anti-TB therapy; only 25.2% received the alternative anti-TB regimens due to adverse drug reactions or drug intolerance. Systemic corticosteroids are recommended as an adjunctive treatment for CNS TB [31], and they can reduce mortality in patients with TBM [32]. However, only 64.5% of the study patients received adjunctive corticosteroid treatment. In addition, a significantly smaller number of patients in the HIV group received this adjunctive agent (p < 0.001). There are several reasons for restricting the prescription of corticosteroids for this particular population. They include co-infection with other opportunistic pathogens, concerns about a severely immunocompromised state, and physician preferences.

Consistent with other studies [17, 33], the present research found fatal outcomes of CNS TB (56/310; 18.1%); however, there have been reports of much higher fatality rates (40–55%) [16, 30]. The immune status, disease severity, MTB load, and drug resistance patterns differed between studies, which may have resulted in the varying rates for fatal cases. This study demonstrated that patients with ID conditions had significantly more fatal outcomes. In addition, having a modified BMRC grade III was strongly associated with overall unfavorable outcomes in HIV patients with TBM, similar to several reports [13, 34, 35]. CNS vascular complications, a high MTB load, and a low percentage of CSF lymphocytes seemed to be associated with unfavorable outcomes like other studies [2, 16, 36]; however, a radiological finding of meningeal enhancement and a low percentage of blood lymphocytes were only two clinical findings strongly associated with the poor outcomes. In addition, the CD4 cell levels seemed to be associated with poor outcomes (Table 3). The decreased CD4 cells relative to a low level of peripheral lymphocytes may cause a poor immune response to eliminate CNS tuberculosis resulting in more invasive disease and fatality. Meanwhile, a reduced score of GCS and a radiological finding of cerebral infarction were independent factors associated with unfavorable outcomes in the NID patients with TBM. Cerebral infarction is a severe vascular complication of TBM, leading to poor outcomes reported by several studies [37–39]. Underuse of adjunctive corticosteroid therapy did not cause more unfavorable outcomes among the HIV group, whereas it was significantly associated with adverse outcomes among the NID group (Table 4). Thus, adjunctive corticosteroid therapy is recommended to reduce adverse outcomes in patients with TBM [32]. In the NID group with TBM, undergoing temporary ventriculostomy was also an independent factor associated with unfavorable outcomes. Our study found that most of the NID patients who underwent the ventriculostomy had reduced GCS scores, delayed time to diagnosis of TBM, more radiological abnormalities, and underuse of adjunctive corticosteroid therapy. These findings might be a causal relationship that increased poor outcomes.

The current work analyzed a large comparative dataset of CNS TB cases with various types of CNS manifestations, and it included substantial subpopulations with different immune statuses. Despite that, the study had some limitations. Firstly, its retrospective design prevented us from assessing essential factors, such as TB contacts, compliance, and long-term follow-up. Patient data were also primarily obtained from the medical chart review, which meant that information on some variables was often incomplete. We could not derive complete information on antiretroviral therapy among the HIV group. As a result, this study still did not know antiretroviral therapy and TBM outcomes. According to the diagnosis criteria of the study, the retrospective chart review restricted a complete evaluation of subclinical and paucibacillary MTB infections in extra-CNS sites and other organs, which resulted in having a more significant proportion of the possible diagnoses (42.6%) than the probable cases (14.8%). Many patients did not undergo CT angiography, MRI, or MR angiography; thus, the radiographic findings and complications related to CNS TB still needed to be fully elucidated. The high percentage of subjects lost to follow-up may have prevented a fair comparison of the treatment outcomes of the groups. Moreover, for individuals with an ID other than HIV infection, the drug resistance profiles and unfavorable outcomes were not included in the outcome analyses due to the low proportion of subjects in each group. Finally, to compound matters, given that the number of subjects included in the study did not reach the calculated sample size, the study might have needed to be more balanced.

## Conclusions

Tuberculous meningitis (TBM) is this study’s most significant proportion of CNS TB. Most of the clinical findings of TBM between the ID and NID patients were indistinguishable. However, it was found that an immunodeficiency disorder, particularly HIV infection, may alter some clinical manifestations, radiological findings, and treatment outcomes. The independent factors associated with overall unfavorable outcomes in the HIV group were having modified BMRC TBM grade III, a finding of meningeal enhancement from imaging studies, and a low percentage of peripheral lymphocytes. Meanwhile, having a low Glasgow Coma Scale score at presentation, a finding of cerebral infarction from imaging studies, underuse of adjunctive corticosteroid therapy, and undergoing temporary ventriculostomy are independent factors associated with overall unfavorable outcomes in the NID group.

### Electronic supplementary material

Below is the link to the electronic supplementary material.

Supplementary Material 1

Supplementary Material 2

## Data Availability

The datasets used and/or analyzed during the current study are available from the corresponding author upon reasonable request.

## List of abbreviations

AFB: Acid-fast bacilli
BMRC: British Medical Research Council
CI: Confidence interval
CNS: Central nervous system
CSF: Cerebrospinal fluid
CT: Computed tomography
HIV: Human immunodeficiency virus
ID: Immunodeficient
LJ: Löwenstein–Jensen
MDR: Multidrug-resistant
MRI: Magnetic resonance imaging
MTB: Mycobacterium tuberculosis
NID: Non-immunodeficient
OR: Odds ratio
PCR: Polymerase chain reaction
SLE: Systemic lupus erythematosus
SOL: Space-occupying lesion
TB: Tuberculosis
TBM: Tuberculous meningitis
