# Supplementary material for: Distinguishing clinical characteristics of central nervous system tuberculosis in immunodeficient and non-immunodeficient individuals: a 12-year retrospective study
Source: Ann Clin Microbiol Antimicrob. 2023 Aug 7;22:69. doi: 10.1186/s12941-023-00615-w (PMC10408106; doi:10.1186/s12941-023-00615-w)
Supplement: Supplementary file 1 — Supplementary Material 1 [file 12941_2023_615_MOESM1_ESM.docx]

**Supplementary Table 1.** Comparison of clinical data, radiological and laboratory findings, and management of tuberculous meningitis between HIV-infected patients with unfavorable outcomes and with favorable outcomes

| **Variable** | **Total**  (n = 78)^a^ | **Unfavorable outcome group**  (n = 31) | **Favorable**  **outcome group**  (n = 47) | ***p-*value** |
| --- | --- | --- | --- | --- |
| Male, n (%) | 55 (70.5) | 20 (64.5) | 35 (74.5) | 0.346 |
| Age, mean ± SD, years | 41.06 ± 11.73 | 41.03 ± 13.54 | 41.09 ± 10.53 | 0.985 |
| BMI, mean ± SD, kg/m² | 20.75 ± 3.91 | 18.49 ± 2.20 | 21.48 ± 4.09 | 0.020 |
| Previous TB diagnosis, n (%) | 25 (32.1) | 11 (35.5) | 14 (29.8) | 0.598 |
| CD_4_ cell level, median (min-max), cells/mm^3^ | 82.5 (1.0-964.0) | 56.0 (1.0-351.0) | 127.0 (1.0-964.0) | 0.026 |
| **Comorbidities**, n (%) | 78 | 31 | 47 |  |
| DM | 2 (2.6) | 0 (0.0) | 2 (4.3) | 0.515 |
| HT | 3 (3.8) | 1 (3.2) | 2 (4.3) | 1.00 |
| Kidney disease | 1 (1.3) | 1 (3.2) | 0 (0.0) | 0.050 |
| Liver disease | 6 (7.7) | 4 (12.9) | 2 (4.3) | 0.208 |
| Lung disease | 12 (15.4) | 5 (16.1) | 7 (14.9) | 1.00 |
| Cancers in remission | 2 (2.6) | 0 (0.0) | 2 (4.3) | 0.515 |
| Others | 8 (10.3) | 4 (12.9) | 4 (8.5) | 0.706 |
| **The final diagnosis of TBM**, n (%) |  |  |  | 0.055 |
| Definite | 25 (32.1) | 12 (38.7) | 13 (27.7) |  |
| Probable | 13 (16.7) | 8 (25.8) | 5 (10.6) |  |
| Possible | 40 (51.3) | 11 (35.5) | 29 (61.7) |  |
| **Concurrent active non-CNS TB**, n (%) |  |  |  |  |
| Lung | 24 (30.8) | 11 (35.5) | 13 (27.7) | 0.464 |
| Lymph node | 10 (12.8) | 4 (12.9) | 6 (12.8) | 1.00 |
| Others | 3 (3.8) | 1 (3.2) | 2 (4.3) | 1.00 |
| Duration of symptoms, median (min-max), days | 10.0 (1.0-84.0) | 14.0 (1.0-84.0) | 10.0 (1.0-84.0) | 0.229 |
| **Clinical manifestations**, n (%) |  |  |  |  |
| Fever | 79 (62.8) | 20 (64.5) | 29 (61.7) | 0.801 |
| Headache | 43 (55.1) | 16 (51.6) | 27 (57.4) | 0.612 |
| Vomiting | 23 (295) | 7 (22.6) | 16 (34.0) | 0.277 |
| Meningeal irritation signs | 54 (69.2) | 24 (77.4) | 30 (63.8) | 0.223 |
| Impaired cognitive function | 38 (48.7) | 19 (61.3) | 19 (40.4) | 0.071 |
| Seizure | 11 (14.1) | 3 (9.7) | 8 (17.0) | 0.511 |
| Hemiparesis | 10 (12.8) | 3 (9.7) | 7 (14.9) | 0.732 |
| Multi-cranial nerve palsy | 2/10 (20.0) | 0/4 (0.0) | 2/6 (33.3) | 0.467 |
| Abnormal movement | 2 (2.6) | 1 (3.2) | 1 (2.1) | 1.00 |
| Impaired sensory systems | 4 (5.1) | 1 (3.2) | 3 (6.4) | 1.00 |
| Bowel and bladder dysfunctions | 3 (3.8) | 2 (6.5) | 1 (2.1) | 0.560 |
| Cerebellar signs | 5 (6.4) | 2 (6.5) | 3 (6.4) | 1.00 |
| Abnormal gait | 3 (3.8) | 1 (3.2) | 2 (4.3) | 1.00 |
| GCS score, mean ± SD | 13.58 ± 2.33 | 12.32 ± 2.70 | 14.40 ± 1.60 | < 0.001 |
| Modified BMRC TBM grade III^b^, n (%) | 10 (12.8) | 8 (25.8) | 2 (4.3) | 0.012 |
| **CNS CT or MRI findings**, n (%) | 78 | 31 | 47 |  |
| Meningeal enhancement | 41 (52.6) | 21 (67.7) | 20 (42.6) | 0.029 |
| Hydrocephalus | 29 (37.2) | 17 (54.8) | 12 (25.5) | 0.009 |
| Cerebral infarction | 27/78 (34.6) | 15/31 (48.4) | 12/47 (25.5) | 0.038 |
| **CSF findings** |  |  |  |  |
| OP, mean ± SD, cmH_2_O | 21.39 ± 9.56 | 22.75 ± 11.31 | 20.55 ± 8.32 | 0.342 |
| WBC count, median (min-max), cells/mm^3^ | 93.0 (1.0-1500) | 85.0 (3.0-1500) | 93.0 (1.0-1350) | 0.361 |
| %neutrophils, mean ± SD, % | 24.12 ± 28.15 | 32.57 ± 30.75 | 18.61 ± 25.15 | 0.043 |
| %lymphocytes, median (min-max), % | 82.0 (1.0-100.0) | 67.0 (4.0-99.0) | 87.0 (1.0-100) | 0.059 |
| Protein, median (min-max), mg/dL | 194.0 (20.0-1668) | 194 (45-1668) | 194.0 (20.0-1000) | 0.291 |
| Glucose, median (min-max), mg/dL | 38.0 (10.0-109.0) | 39.5 (10.0-98.0) | 38.0 (12.0-109.0) | 0.181 |
| CSF to plasma glucose ratio, median (min-max) | 0.31 (0.08-0.57) | 0.32 (0.08-0.57) | 0.31 (0.1-0.56) | 0.415 |
| AFB positive, n (%) | 2/77 (2.6) | 2/30 (6.7) | 0/47 (0.0) | 0.149 |
| Direct PCR MTB positive, n (%) | 12/77 (15.6) | 8/30 (26.7) | 4/47 (8.5) | 0.051 |
| MTB culture positive, n (%) | 27/77 (35.1) | 12/30 (40.0) | 15/47 (31.9) | 0.468 |
| **Initial hematologic testing** |  |  |  |  |
| Hb, mean ± SD, g/dL | 10.86 ± 2.11 | 10.77 ± 2.38 | 10.92 ± 1.95 | 0.763 |
| Hct, mean ± SD, percent | 32.95 ± 6.22 | 32.78 ± 6.56 | 33.06 ± 1.95 | 0.847 |
| WBC count, median (min-max), cells/mm^3^ | 6800 (1140-19070) | 7320 (1140-19070) | 6240 (1200-16680) | 0.198 |
| %neutrophils, mean ± SD, % | 69.88 ± 16.52 | 75.62 ± 13.35 | 66.09 ± 17.43 | 0.012 |
| %lymphocytes, median (min-max), % | 15.5 (1.0-60.0) | 10.7 (1.0-34.0) | 21.6 (3.9-60.0) | < 0.001 |
| BUN, median (min-max), mg/dL | 13.0 (0.8-131.0) | 12.0 (6.6-131.0) | 13.0 (0.8-24.1) | 0.846 |
| Cr, median (min-max), mg/dL | 0.8 (0.3-2.71) | 0.7 (0.34-2.71) | 0.84 (0.3-1.9) | 0.034 |
| AST, median (min-max), U/L | 39.5 (12.0-1056) | 41.0 (13.0-1056) | 34.0 (12.0-466.0) | 0.377 |
| ALT, median (min-max), U/L | 24.0 (6.0-516.0) | 25.0 (6.0-516.0) | 23.0 (7.0-436.0) | 0.910 |
| ALP, median (min-max), IU/L | 80.0 (25.0-698.0) | 86.0 (25.0-452.0) | 80.0 (43.0-698.0) | 0.802 |
| Albumin, mean ± SD, g/dL | 3.29 ± 0.78 | 3.11 ± 0.80 | 3.42 ± 0.75 | 0.084 |
| Sodium level, mean ± SD, mmol/L | 131.69 ± 6.09 | 130.71 ± 6.78 | 132.34 ± 5.52 | 0.247 |
| **Anti-TB drug susceptibility testing**^c^, n (%) |  |  |  |  |
| Performed | 27 (34.6) | 12 (38.7) | 15 (31.9) | 0.537 |
| Not performed | 51 (65.4) | 19 (61.3) | 32 (68.1) |  |
| Fully susceptible | 16/27 (59.3) | 7/12 (58.3) | 9/15 (60.0) | 1.00 |
| Isoniazid monoresistance | 4/27 (14.8) | 0/12 (0.0) | 4/15 (26.7) | 0.106 |
| Rifampin monoresistance | 0/27 (0.0) | 0/12 (0.0) | 0/15 (0.0) | - |
| Pyrazinamide monoresistance | 1/27 (3.7) | 1/12 (8.3) | 0/15 (0.0) | 0.444 |
| Multidrug resistance | 5/27 (4.5) | 3/12 (25.0) | 2/15 (13.3) | 0.628 |
| **Initial anti-TB treatment**, n (%) |  |  |  | 0.933 |
| Standard combination regimen^d^ | 60 (76.9) | 24 (31) | 36 (76.6) |  |
| Alternative or modified regimen^e^ | 18 (23.1) | 7 (22.6) | 11 (23.4) |  |
| Adjunctive corticosteroid therapy, n (%) | 40 (51.3) | 14 (45.2) | 26 (55.3) | 0.380 |
| **Surgical interventions**, n (%) | 78 | 31 | 47 |  |
| Temporary ventriculostomy | 1 (1.3) | 1 (3.2) | 0 (0.0) | 0.397 |

Abbreviations: %lymphocyte, percentage of lymphocytes; %neutrophil, percentage of neutrophils; AFB, acid-fast bacilli; ALP, alkaline phosphatase; ALT, alanine transaminase; AST, aspartate transaminase; BMI, body mass index; BMRC, British Medical Research Council; BUN, blood urea nitrogen; CN, cranial nerve; CNS, central nervous system; Cr, creatinine; CSF, cerebrospinal fluid; CT, computed tomography; DM, diabetes mellitus; GCS, Glasgow Coma Scale; Hb, hemoglobin; Hct, hematocrit; HIV, human immunodeficiency virus; HT, hypertension; MRI, magnetic resonance imaging; MTB, *Mycobacterium tuberculosis*; OP, opening pressure; PCR, polymerase chain reaction; TB, tuberculosis; TBM, tuberculous meningitis; WBC, white blood cell

^a^ Excluding 45 TBM patients with unknown outcomes

^b^ Defined as disease severity with a GCS score ≤ 10

^c^ Anti-TB drug susceptibility was tested by the agar proportion method and the determination of the mycobacterial growth ratio of drug-containing and drug-free broths.

^d^ A combination of anti-TB agents, namely, isoniazid, rifampin, pyrazinamide, and ethambutol

^e^ A standard anti-TB regimen was switched or modified to alternative agents due to adverse reactions, drug intolerance, or drug allergy.
