# Supplementary material for: Distinguishing clinical characteristics of central nervous system tuberculosis in immunodeficient and non-immunodeficient individuals: a 12-year retrospective study
Source: Ann Clin Microbiol Antimicrob. 2023 Aug 7;22:69. doi: 10.1186/s12941-023-00615-w (PMC10408106; doi:10.1186/s12941-023-00615-w)
Supplement: Supplementary file 2 — Supplementary Material 2 [file 12941_2023_615_MOESM2_ESM.docx]

**Supplementary Table 2.** Comparison of clinical data, radiological and laboratory findings, and management of tuberculous meningitis between non-immunodeficient patients with unfavorable outcomes and favorable outcomes

| **Variable** | **Total**  (n = 107) | **Unfavorable outcome group**  (n = 28) | **Favorable**  **outcome group**  (n = 79) | ***p-*value** |
| --- | --- | --- | --- | --- |
| Male, n (%) | 64 (59.8) | 18 (64.3) | 46 (58.2) | 0.574 |
| Age, mean ± SD, years | 47.75 ± 20.68 | 57.96 ± 19.98 | 44.13 ± 19.81 | 0.002 |
| BMI, mean ± SD, kg/m² | 20.20 ± 3.40 | 19.78 ± 4.58 | 20.3 2± 3.05 | 0.575 |
| Previous TB diagnosis, n (%) | 23 (21.5) | 7 (25.0) | 16 (20.3) | 0.599 |
| **Comorbidities**, n (%) | 107 | 28 | 79 |  |
| DM | 10 (9.3) | 4 (14.3) | 6 (7.6) | 0.285 |
| HT | 22 (20.6) | 11 (39.3) | 11 (13.9) | 0.004 |
| Kidney disease | 5 (4.7) | 4 (14.3) | 1 (1.3) | 0.016 |
| Liver disease | 5 (4.7) | 0 (0.0) | 5 (6.3) | 0.323 |
| Heart disease | 7 (6.5) | 4 (14.3) | 3 (3.8) | 0.075 |
| Lung disease | 10 (9.3) | 3 (10.7) | 7 (8.9) | 0.719 |
| Cancers in remission | 4 (3.7) | 2 (7.1) | 2 (2.5) | 0.280 |
| Others | 16 (15.0) | 8 (28.6) | 8 (10.1) | 0.029 |
| **The final diagnosis of TBM**, n (%) |  |  |  | 0.687 |
| Definite | 54 (50.5) | 16 (57.1) | 38 (48.1) |  |
| Probable | 38 (35.5) | 9 (32.1) | 29 (36.7) |  |
| Possible | 15 (14.0) | 3 (10.7) | 12 (15.2) |  |
| **Concurrent active non-CNS TB**, n (%) |  |  |  |  |
| Lung | 38 (35.5) | 13 (46.4) | 25 (31.6) | 0.160 |
| Pleura | 1 (0.9) | 1 (3.6) | 0 (0.0) | 0.262 |
| Lymph node | 4 (3.7) | 2 (7.1) | 2 (2.5) | 0.280 |
| Others | 11 (10.3) | 4 (14.3) | 7 (8.9) | 0.473 |
| Duration of symptoms, median (min-max), days | 14.0 (1.0-140.0) | 14.0 (3.0-56.0) | 14.0 (1.0-140.0) | 0.292 |
| **Clinical manifestations**, n (%) |  |  |  |  |
| Fever | 65 (60.7) | 16 (57.1) | 49 (62.0) | 0.649 |
| Headache | 60 (56.1) | 9 (32.1) | 51 (64.6) | 0.003 |
| Vomiting | 36 (33.6) | 6 (21.4) | 30 (38.0) | 0.111 |
| Meningeal irritation signs | 67 (62.6) | 16 (57.1) | 51 (64.6) | 0.486 |
| Impaired cognitive function | 52 (48.6) | 12 (42.9) | 40 (50.6) | 0.479 |
| Seizure | 14 (13.1) | 3 (10.7) | 11 (13.9) | 1.00 |
| Hemiparesis | 5 (4.7) | 0 (0.0) | 5 (6.3) | 0.323 |
| Multi-cranial nerve palsy | 6/20 (30.0) | 2/6 (33.3) | 4/14 (28.6) | 1.00 |
| Abnormal movement | 2 (1.9) | 1 (3.6) | 1 (1.3) | 0.457 |
| Impaired sensory systems | 4 (3.7) | 1 (3.6) | 3 (3.8) | 1.00 |
| Bowel and bladder dysfunctions | 4 (3.7) | 2 (7.1) | 2 (2.5) | 0.280 |
| Cerebellar signs | 7 (6.5) | 3 (10.7) | 4 (5.1) | 0.375 |
| Abnormal gait | 2 (1.9) | 0 (0.0) | 2 (2.5) | 1.00 |
| GCS score, mean ± SD | 13.62 ± 2.42 | 11.50 ± 3.11 | 14.37 ± 1.55 | < 0.001 |
| Modified BMRC TBM grade III^b^, n (%) | 8 (7.5) | 6 (21.4) | 2 (2.5) | 0.004 |
| **CNS CT or MRI findings**, n (%) | 105 | 14 | 77 |  |
| Meningeal enhancement | 54 (51.4) | 14 (50.0) | 40 (51.9) | 0.860 |
| Hydrocephalus | 34 (32.4) | 12 (42.9) | 22 (28.6) | 0.167 |
| Cerebral infarction | 23/104 (22.1) | 10/28 (35.7) | 13/76 (17.1) | 0.061 |
| **CSF findings** |  |  |  |  |
| OP, mean ± SD, cmH_2_O | 21.91 ± 8.90 | 21.24 ± 9.64 | 22.15 ± 8.68 | 0.650 |
| WBC count, median (min-max), cells/mm^3^ | 124 (2.0-2800) | 67.0 (3.0-1500) | 159.50 (2.0-2800) | 0.009 |
| %neutrophils, mean ± SD, % | 24.48 ± 25.74 | 29.54 ± 26.78 | 22.68 ± 25.31 | 0.246 |
| %lymphocytes, median (min-max), % | 82.0 (2.0-100.0) | 67.0 (11.0-100.0) | 84.0 (2.0-100) | 0.348 |
| Protein, median (min-max), mg/dL | 188.0 (46.0-4482) | 185 (74-2595) | 189.5 (46.0-4482) | 0.479 |
| Glucose, median (min-max), mg/dL | 35.0 (1.0-141.0) | 35.0 (3.0-92.0) | 34.0 (1.0-141.0) | 0.826 |
| CSF to plasma glucose ratio, median (min-max) | 0.31 (0.01-0.78) | 0.31 (0.11-0.50) | 0.30 (0.01-0.78) | 0.889 |
| AFB positive, n (%) | 4/105 (3.8) | 2/27 (7.4) | 2/78 (2.6) | 0.272 |
| Direct PCR MTB positive, n (%) | 11/105 (10.5) | 3/27 (11.1) | 8/78 (10.3) | 1.00 |
| MTB culture positive, n (%) | 49/105 (46.7) | 16/27 (59.3) | 33/78 (42.3) | 0.128 |
| **CNS tissue pathology examination**, n (%) |  |  |  |  |
| AFB positive | 1/7 (14.3) | 1/1 (100) | 0/6 (0.0) | 0.143 |
| Direct PCR MTB positive | 2/6 (33.3) | 1/1 (100) | 1/5 (20.0) | 0.333 |
| MTB culture positive | 2/6 (33.3) | 0/1 (0.0) | 2/5 (40.0) | 1.00 |
| **Initial hematologic testing** |  |  |  |  |
| Hb, mean ± SD, g/dL | 12.00 ± 1.97 | 11.87 ± 1.92 | 12.05 ± 2.00 | 0.686 |
| Hct, mean ± SD, percent | 36.25 ± 5.46 | 35.91 ± 5.40 | 36.37 ± 5.52 | 0.705 |
| WBC count, median (min-max), cells/mm^3^ | 8980 (3860-25340) | 7770 (4100-22300) | 9510 (3860-25340) | 0.278 |
| %neutrophils, mean ± SD, % | 77.02 ± 10.47 | 78.91 ± 9.55 | 76.35 ± 10.76 | 0.268 |
| %lymphocytes, median (min-max), % | 11.1 (2.3-39.9) | 9.90 (2.50-23.0) | 12.00 (2.39-39.90) | 0.114 |
| BUN, median (min-max), mg/dL | 12.50 (2.20-95.0) | 20.7 (8.0-80.0) | 12.1 (2.20-95.0) | 0.003 |
| Cr, median (min-max), mg/dL | 0.73 (0.3-9.3) | 0.81 (0.49-9.30) | 0.70 (0.3-4.70) | 0.017 |
| AST, median (min-max), U/L | 26.0 (9.0-167) | 37.0 (10.0-98.0) | 23.0 (9.0-167.0) | 0.033 |
| ALT, median (min-max), U/L | 23.0 (3.0-338.0) | 24.0 (3.0-80.0) | 22.0 (7.0-388.0) | 0.593 |
| ALP, median (min-max), IU/L | 75.0 (36.0-428.0) | 79.5 (37.0-267.0) | 73.0 (36.0-428.0) | 0.502 |
| Albumin, mean ± SD, g/dL | 3.52 ± 0.66 | 3.19 ± 0.62 | 3.63 ± 0.65 | 0.003 |
| Sodium level, mean ± SD, mmol/L | 130.92 ± 7.15 | 130.21 ± 6.06 | 131.17 ± 7.52 | 0.548 |
| **Anti-TB drug susceptibility testing**^c^, n (%) |  |  |  |  |
| Performed | 55 (51.4) | 16 (57.1) | 39 (49.4) | 0.479 |
| Not performed | 52 (48.6) | 12 (42.9) | 40 (50.6) |  |
| Fully susceptible | 46/55 (83.6) | 11/16 (68.8) | 35/39 (89.7) | 0.103 |
| Isoniazid monoresistance | 3/55 (5.5) | 2/16 (12.5) | 1/39 (2.6) | 0.200 |
| Rifampin monoresistance | 1/55 (1.8) | 0/16 (0.0) | 1/39 (2.6) | 1.00 |
| Pyrazinamide monoresistance | 4/55 (7.3) | 2/16 (12.5) | 2/39 (5.1) | 0.571 |
| Multidrug resistance | 0/55 (0.0) | 0/16 (0.0) | 0/39 (0.0) | - |
| **Initial anti-TB treatment**, n (%) |  |  |  |  |
| Standard combination regimen^d^ | 84 (78.5) | 23 (82.1) | 61 (77.2) | 0.585 |
| Alternative or modified regimen^e^ | 23 (21.5) | 5 (17.9) | 18 (22.8) |  |
| Adjunctive corticosteroid therapy, n (%) | 76 (71.0) | 15 (53.6) | 61 (77.2) | 0.018 |
| **Surgical interventions**, n (%) | 107 | 28 | 79 |  |
| Temporary ventriculostomy | 7 (6.5) | 5 (17.9) | 2 (2.5) | 0.013 |
| Ventriculoperitoneal shunt | 1 (0.9) | 0 (0.0) | 1 (1.3) | 1.00 |

Abbreviations: %lymphocyte, percentage of lymphocytes; %neutrophil, percentage of neutrophils; AFB, acid-fast bacilli; ALP, alkaline phosphatase; ALT, alanine transaminase; AST, aspartate transaminase; BMI, body mass index; BMRC, British Medical Research Council; BUN, blood urea nitrogen; CN, cranial nerve; CNS, central nervous system; Cr, creatinine; CSF, cerebrospinal fluid; CT, computed tomography; DM, diabetes mellitus; GCS, Glasgow Coma Scale; Hb, hemoglobin; Hct, hematocrit; HT, hypertension; MRI, magnetic resonance imaging; MTB, *Mycobacterium tuberculosis*; OP, opening pressure; PCR, polymerase chain reaction; TB, tuberculosis; TBM, tuberculous meningitis; WBC, white blood cell

^a^ Excluding 28 TBM patients with unknown outcomes

^b^ Defined as disease severity with a GCS score ≤ 10

^c^ Anti-TB drug susceptibility was tested by the agar proportion method and the determination of the mycobacterial growth ratio of drug-containing and drug-free broths.

^d^ A combination of anti-TB agents, namely, isoniazid, rifampin, pyrazinamide, and ethambutol

^e^ A standard anti-TB regimen was switched or modified to alternative agents due to adverse reactions, drug intolerance, or drug allergy.
